# Indoor Air-Quality Data-Monitoring System: Long-Term Monitoring Benefits

**DOI:** 10.3390/s19194157

**Published:** 2019-09-25

**Authors:** Shengjing Sun, Xiaochen Zheng, Javier Villalba-Díez, Joaquín Ordieres-Meré

**Affiliations:** 1 ETSII, Universidad Politécnica de Madrid, José Gutiérrez Abascal 2, 28006 Madrid, Spain; xiaochen.zheng@epfl.ch (X.Z.); j.ordieres@upm.es (J.O.-M.); 2 Exposure, Epidemiology, and Risk Program, Department of Environmental Health, Harvard T.H. Chan School of Public Health, Boston, MA 02115, USA; 3 School of Engineering, Institute of Mechanical Engineering. École polytechnique fédérale de Lausanne, 1015 Lausanne, Switzerland; 4 Fakultaet fuer Management und Vertrieb, Campus Schwäbisch-Hall, Hochschule Heilbronn, 74081 Heilbronn, Germany; javier.villalba-diez@hs-heilbronn.de; 5 ETSIInformática, Department of Artificial Intelligence, Universidad Politécnica de Madrid, Calle de los Ciruelos, Boadilla del Monte, 28660 Madrid, Spain

**Keywords:** long-term monitoring benefits, indoor air quality, low cost, occupational safety and health, industry 4.0, IOTA tangle

## Abstract

Indoor air pollution has been ranked among the top five environmental risks to public health. Indoor Air Quality (IAQ) is proven to have significant impacts on people’s comfort, health, and performance. Through a systematic literature review in the area of IAQ, two gaps have been identified by this study: short-term monitoring bias and IAQ data-monitoring solution challenges. The study addresses those gaps by proposing an Internet of Things (IoT) and Distributed Ledger Technologies (DLT)-based IAQ data-monitoring system. The developed data-monitoring solution allows for the possibility of low-cost, long-term, real-time, and summarized IAQ information benefiting all stakeholders contributing to define a rich context for Industry 4.0. The solution helps the penetration of Industrial Internet of Things (IIoT)-based monitoring strategies in the specific case of Occupational Safety Health (OSH). The study discussed the corresponding benefits OSH regulation, IAQ managerial, and transparency perspectives based on two case studies conducted in Spain.

## 1. Introduction

Indoor air pollution is a leading environmental risk, which affects people’s working performance, comfort, health, and well-being [1,2,3]. People spend around 90% of their time indoors, and human exposure to indoor air pollutants may occasionally be more than 100 times higher than outdoor pollutant levels [4]. Exposure to poor indoor air is a significant cause of productivity loss, for the U.S., as productivity decreases 0.5–5% per workplace, generating a loss of 20 to 200 billion US dollars annually [5].

Indeed, exposure to poor indoor air also increases numerous adverse health problems, such as nausea, headaches, skin irritation, sick building syndrome, kidney failure, and even cancer [1,3,6]. The World Health Organization (WHO) estimates that 700,000 people per year die from poor breathing conditions [7]. Therefore, IAQ, has a significant impact on people’s comfort, health, and performance.

IAQ have been investigated by research community and practitioners. However, two important gaps are identified by a systematic literature review. Details of literature review method and results are presented in Appendix A. The first gap is that long-term data-monitoring is lacking in the IAQ research community and in practice. The second gap is that IAQ data monitoring solution is hard to operate, expensive, and lacks transparency in terms of Occupational Safety Health (OSH) management.

**Gap I**. In most research work, sampling time period is relatively short term, and even sometimes lasts for less than one hour. Measurements were made based on assumption the IAQ conditions are the same during the year [3,8,9]. Several studies have shown that seasonal variation or non-heating and heating time period differences [10,11,12], considering IAQ variation by season climate change and human heating behavior. However, those studies only took a short time sampling each season. As IAQ varies from time to time due to changes in working conditions, human activity, and weather conditions etc., short-term or seasonal sampling could not cover all kinds of variations. Therefore, long-term monitoring becomes a need in the research community.

In addition to the research dimension, the practical applications, it is increasingly becoming important to gather indoor working conditions to evaluate and minimize adverse health problems. To ensure OSH, environmental regulatory agencies, e.g., Occupational Safety and Health Administrations (OSHA) and local authorities have developed monitoring strategies to assess employee exposure to indoor pollutants. Generally, the monitoring reference period is a short-term basis. A Short-Term Exposure Limit (STEL) is a term used in occupational health, industrial hygiene, and toxicology, and it is regularly adopted to be 15 min.

Long-term monitoring is challenging for environmental regulatory agencies considering instrument calibration, labor time, and costs. In regular monitoring strategies, the professional determination method is used to measure indoor contaminant level. For measuring Volatile Organic Components (VOCs), is to collect air samples, either based on whole air samples in SUMMA passivated stainless steel canisters or on solid adsorbent tubes. Subsequently, the VOCs are separated by gas chromatography and measured by mass-selective detector or multidetector techniques in a remote laboratory basis [13,14]. Due to obstacles in sampling pump technology, professional particulate matter meters such as personal environmental monitor (PEM) with Leland Legacy pump and sioutas personal cascade impactor sampler, only allow maximal 24-hour sampling [15]. Therefore, although those determination methods are accurate, in practice, it will not be possible to have a long-term monitoring strategy with professional instruments.

There are specific measurements considering different episodes, such as an eight-hour working period, night and day cycles, and seasonal variations. However, indoor working conditions vary greatly over time, and on spot short-term sampling or measurement in specific episodes could fail to provide a holistic assessment of the working environment. Long-term monitoring becomes a demand in the workplace and real applications such as OSH management.

**Gap II**. IAQ data-monitoring solution challenges are presented into two aspects. One challenge is that IAQ data sampling/collection is expensive, complex to customize and operate, and professional expertise dependency. To measure multiple pollutants, various devices should be bought from different manufactures [3,10,12]. For example, Aeroqual 200 to measure nitrogen dioxide (NO_2_) and Total Volatile Organic Components (TVOC); Extech VPC300 to measure particulate matter (PM); htV-M to measure formaldehyde (HCHO); Q-Trak to measure carbon monoxide (CO). In addition, diffusive or passive samplers should be prepared and analyzed by chemical domain experts, and frequently replaced with new ones due to limited equipment lifespan [8,11,12], which is complicated to operate and time and labor consuming. Moreover, since different pollutants are measured with different instruments, the various collected pollutant data is complex to manage and process. Another challenge is that IAQ data sampling/collection lacks data sharing in terms of IAQ transparency to all stakeholders. IAQ transparency is vital for managers to regulate working conditions, because IAQ affects employee’s productivity, comfort, and health [2]. IAQ transparency is significant for employees to enhance worker empowerment. However, few data-monitoring solutions consider IAQ data- sharing from a transparent point of view.

**Figure 1:**
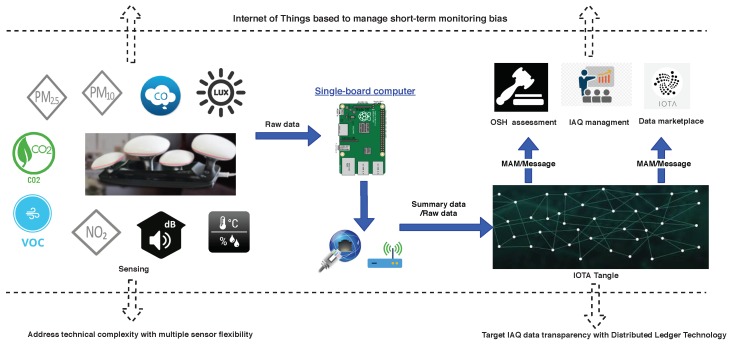
Graphical Abstract. IAQ: Indoor Air Quality; MAM: Masked Authenticated Messaging; OSH: Occupational Safety Health.

As the graphical abstract shows in Figure 1, the research presented in this study will address those gaps by proposing an IoT and DLT based IAQ data-monitoring system. The system is a light, low-cost, long-term solution enabled by blooming development of IoT and sensor technology, and it does enable customization because of users can freely choose pollutant sensors by a simple plug-in. It supports multiple pollutants data-monitoring, which will facilitate research community and practitioners for long-term and integrated multiple pollutants data-monitoring solutions. The main contribution from the paper is not only the hardware layer involved, although it provides a solution ahead of what it is possible to find in the market these days, mainly because of the provided flexibility but also because of the data released over a public tangle in both ways, consolidated summaries as well as stream data flow, which overcomes the private clouds that providers used to adopt. Such approach is hard to handle in Europe regarding the General Data Protection Regulation (GDPR).

The paper promotes an open view of interaction from different layers of application, which enables different kinds of relationships and actually becomes kind of referential framework. The promoted system, which fits into the framework, applies IOTA distributed ledger techniques (concept introduced in Section 2.1) to enable data sharing, which benefits all stakeholders. It provides flexibility and low-cost, real-time, and summarized IAQ information to managers, aligns IAQ transparency to worker empowerment, and enables other benefits related to improving environments in the workplace. The relevant benefits are discussed and presented by two case studies conducted in Spain, from perspectives of OSH assessment, regulating working conditions, OSH transparency and data sharing with IOTA distributed ledger techniques.

A significant point highlighted by this paper is that although the paradigm of Industry 4.0 is being widely adopted by many industries in different sectors, there is a lack of penetration of these technologies in the specific case of the OSH [16]. Actually, the contribution from the IIoT can be considered as very significant because it can help in changing the implemented monitoring strategies. A contribution in this line is essentially the way adopted in in this paper.

## 2. Materials and Methods

### 2.1. Framework

**Figure 2:**
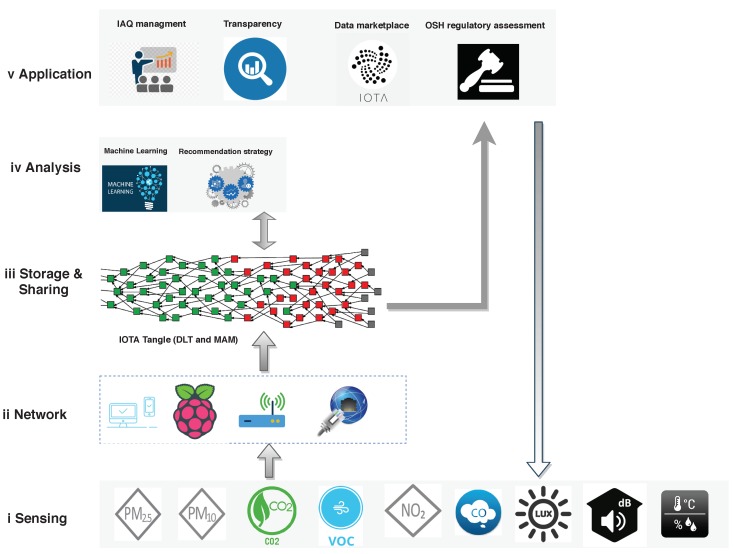
IAQ data-monitoring application framework supported by Internet of Things (IoT) and Distributed Ledger Technology (DLT). IAQ: Indoor Air Quality; MAM: Masked Authenticated Messaging; OSH: Occupational Safety Health.

The framework of the proposed data-monitoring architecture is presented in Figure 2. It is composed of five layers, including the sensing layer, network layer, IOTA-based data storage & sharing layer, data analysis layer, and application layer. (i) The sensing layer is the perception layer, which is the lowest layer of the conventional architecture of IoT. It contains different sensors for sensing and gathering IAQ information; (ii) The network layer is responsible for connecting to other smart things, network devices, and servers. It also facilitates transmitting and processing sensor data; (iii) The storage & sharing layer is based on DLT, the IOTA Tangle in this case, which supports secure and tamper-resistant data storage and sharing. The raw IAQ data can be shared through IOTA Tangle and in return the data publisher can receive monetary or other types of benefits. Moreover, the IAQ data analysis results, e.g., recommendation strategies, can also be shared through IOTA Tangle to help enhance regulation assessment; (iv) The analysis layer receives data from previous layers and use certain data processing techniques and machine learning models, to extract information and knowledge or even further means of wisdom; (v) The application layer is responsible for delivering specific services to corresponding users. It defines various scenarios in which the IoT and DLT can be deployed, for example, information transparency, IAQ management and assessment, gaining economic benefits for data providers. The proposed framework can also address low-cost sensor accuracy issue, as it supports persistently data aligning with OSH regulatory assessment conducted by high reliable professional instrument. The main enabling technologies involved in this framework are introduced as follows:

- **IoT**. The term "Internet of things" was coined by Kevin Ashton of Procter & Gamble in 1999, when he viewed Radio-frequency identification (RFID) as essential to the IoT, allowing computers to manage all individual things (all existing things). Presently, the IoT concept is that the pervasive presence of a variety of things or objects—such as RFID tags, sensors, actuators, mobile phones, etc.
- **Low-cost IoT-based sensing**. The low-cost IoT sensors enable the use of wireless communications and computing for interacting with the physical world. The relevant sensors could sense indoor environmental parameters such as IAQ, comfort, lighting, and acoustic conditions. Several systems [17,18,19] have been developed for monitoring indoor environmental conditions with low-cost sensors. The data quality generated by these sensors are often of questionable. The performance of different low-cost air-quality sensors vary from unit to unit, spatially and temporally, as it relies on different algorithms, the meteorological conditions and atmospheric composition [20]. The IAQ data-monitoring platform implemented in this study is low-cost sensor-based considering that high accuracy is not the top requirement for the targeted applications of this study. Instead, this platform is developed for purposes such as awareness raising and recommendation of sampling period selection for OSH legal compliance, which only demand the pollution level on a coarse scale. In addition, as shown in Figure 2, the accuracy of the proposed platform can be improved through data adjustment with professional instrument at each OSH regulatory spot-check in long periods, just by observing potential bias or sensor saturation.
- **Network**. The network e.g., IoT gateway, bridges sensor networks with the traditional communication networks. It settles the heterogeneity between various sensor networks, mobile communication networks, and the Internet (all computer networks) [21,22]. A single-board computer (SBC), such as Raspberry Pi, could provide low-cost and efficient gateway services based on emerging IoT standards.
- **DLT**. Blockchain, as the first DLT, was invented by Satoshi Nakamoto in 2008 to serve as the public transaction ledger of the cryptocurrency Bitcoin [23]. The main component of DLT is a distributed ledger, which is used as a distributed database maintained by a consensus protocol run by nodes in a peer-to-peer network. This consensus protocol replaces a central administrator, since all peers contribute to maintaining the integrity of the database [24]. With a decentralized and consensus-driven nature, DLT could provide reliable solutions, such as blockchain [23], Ethereum [25] and IOTA Tangle [26], to enable secure and tamper-resistant data storage and sharing.
- **IOTA and the Tangle**. IOTA is a tangle-based cryptocurrency designed specifically for the IoT industry. The IOTA tangle naturally succeeds the blockchain as its next evolutionary step by overcoming some of its fundamental limitations, such as scalability, high transaction fees, and vulnerability to quantum attack [26]. The main feature of the tangle is that it uses a Directed Acyclic Graph (DAG) for storing transactions instead of sequential blocks. In the Tangle, users need to perform a small amount of computational work to approve two previous transactions to issue a new transaction. This new transaction will be validated by subsequent transactions [27].
- **Masked Authenticated Messaging (MAM)**. The main data communication protocol used for data sharing in IOTA is MAM. It enables clients to emit and access an encrypted data streams over the IOTA Tangle, regardless of the size or cost of a device [28]. MAM uses channels (Public/Private/Restricted) for message spreading. IOTA users can create a channel and publish a message of any size, at any time. A small amount of proof-of-work is required to allow the data to propagate through the network and to prevent spamming. Other users can subscribe to this channel through its address, and receive a message that is published by the channel owner.

The framework is also a functional guide following presented research work, for better understanding the IoT and DLT-based IAQ data-monitoring system and its long-term monitoring benefits.

### 2.2. Standards and Guidelines for OSH Assessment

The US National Institute for Occupational Safety and Health (NIOSH) defined a recommended exposure limit (REL) for hundreds of workplace chemical contaminants [29]. For NIOSH RELs, a time-weighted average (TWA) concentration was measured for up to a 10 h workday during a 40 h workweek. They also define STEL as a TWA exposure that should not be exceeded at any time during a workday. For a ceiling REL, it is the ceiling value which should not be exceeded at any time. The STEL is as a legal limit for the exposure of an employee to a chemical substance. For chemicals, STEL assessments last for 15 min and are expressed in parts per million (ppm), or sometimes in milligrams per cubic meter (mg/m^3^). Table 1 lists a couple of indoor chemical pollutants and their STEL, presented in NIOSH. We also list an averaging period of 24 h Threshold Limit Value (TLV) for PM referencing from EPA [30] and European Union (EU) air-quality standards [31], because NIOSH only provides TLV for a total particulate 10 mg/m^3^ 8 h TWA.

Considering the variations of workplace concentrations that originate from work patterns, processes (batch production or continuous process), human activity, and meteorological variations, several samples are required for the whole air-quality testing procedure. As shown in Table 2, the sampling duration and its related number of samples are presented, introduced by standard EU BS EN 689:1996.

### 2.3. IoT and DLT-Based Data-Monitoring System

**Figure 3:**
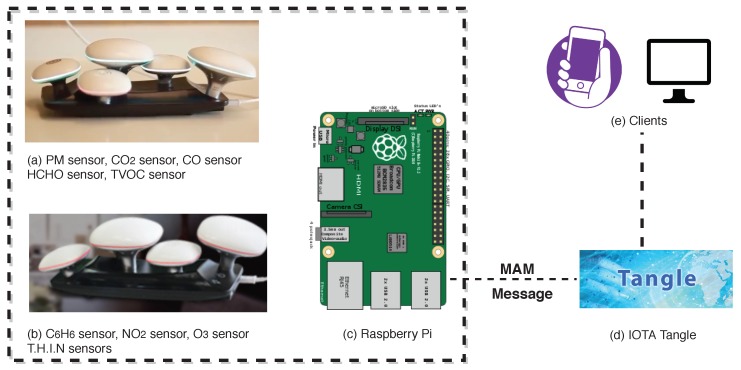
Deployment of Indoor Air-Quality (IAQ) data-monitoring system, enabling different type of clients. MAM: Masked Authenticated Messaging.

The research, unifying the proposed framework, has designed an IoT and DLT-based IAQ data-monitoring system (Figure 3), which is developed using kagoo devices manufactured by Circulate (Figure 3a,b), Raspberry Pi (Figure 3c), and IOTA Tangle (Figure 3d).

The kagoo devices (Figure 3a,b were adopted as the sensing layer. Several indoor environmental conditions are measured with those devices, including air quality, acoustic conditions, lighting, and thermal comfort. Nine sensors, particulate matter (PM), formaldehyde (HCHO), TVOC, benzene (C_6_H_6_), carbon dioxide (CO_2_), carbon monoxide (CO), ozone (O_3_), nitrogen dioxide (NO_2_), and T.H.I.N (Temperature & Humidity & Illumination & Noise), are used to measure indoor conditions. Those sensors can be freely selected and plugged into an island, where five maximal sensors are enabled. The list of mushroom sensors are presented in Table 3.

The SBC Raspberry Pi (Figure 3c) was used as the network layer. A python program was run at Raspberry Pi to collect data measurements from sensors, parse data measurements, and calculate raw data measurement and statistic summaries. The raw data is collected with a frequency of 1 min. Usually, data measurements are stored every 1 min or 5 min in most IAQ monitoring devices [32,33]. The study took 1 min since smaller granularity data could be collected for data analysis. The relevant source code could be found on GitLab [34].

The continuous collected raw data is transmitted to IOTA Tangle through MAM communication protocol. The transmitted data will be broadcast in a streaming channel. Any IOTA user who knows the address of the channel (and the private key in case the channel is restricted) can consume the IAQ data.

The periodical statistic summary, such as 15 min (STEL basis) average indoor pollutant concentration, is uploaded and stored to IOTA Tangle as a message. Through different clients, all the users can review or use the real-time and long-term periodic statistic summary results, which could be applied for OSH regular assessment, IAQ management, and employee transparency for worker empowerment. For instance, when the organization needs to be OSH assessed, they can share with the regulatory authorities the data access from a certain time. Afterward, the regulatory agencies will be able to fetch data streams (e.g., statistic summary data report) for further assessment. Considering low-cost sensor’s accuracy constrains, as the initial application, those data streams could be taken as pre-assessment to ensure spot-checking is conducted efficiently and effectively. Indeed, larger populations could assess the data as scalability was considered to be a capability in the system.

### 2.4. Case Studies

Two case studies were conducted in Spain. The choice of location selection was mainly based on availability of sites; however, the location of each industrial building was carefully examined regarding the influence of the surroundings, urban/rural area, and green zone/heave traffic. Regarding their building structure, the selected locations were representative of the building stock of the country in terms of typology, construction techniques, and age. Site 1 is an advertising workshop located at a warehouse area next to a highway. Site 2 is a steel processing plant located at a rural area near a busy road, where the office section is monitored. The office represents the most common working environment and the advertising workshop represents a less common but with possible higher pollution environment. The site characteristics are listed in Table 4.

The chemical and physical parameters measured were PM2.5, PM10, HCHO, TVOC, C_6_H_6_, CO_2_, CO, O_3_, NO_2_, temperature, humidity, illumination, and noise. The data-monitoring system was placed near worker activity area, with a height of 1.5 m above the ground. On Site 1, the data-monitoring period was from 15 October 2018 to 15 November 2018. On Site 2, the office section is from 7 December 2018 to 11 January 2019. 24 h with granularity 1-minute data measurements were collected inside two sites. All collected data are open access for researchers in OSF (https://osf.io/t6rp8/).

## 3. Results and Discussion

### 3.1. Long-term Monitoring Benefits for OHS Assessment

**Figure 4:**
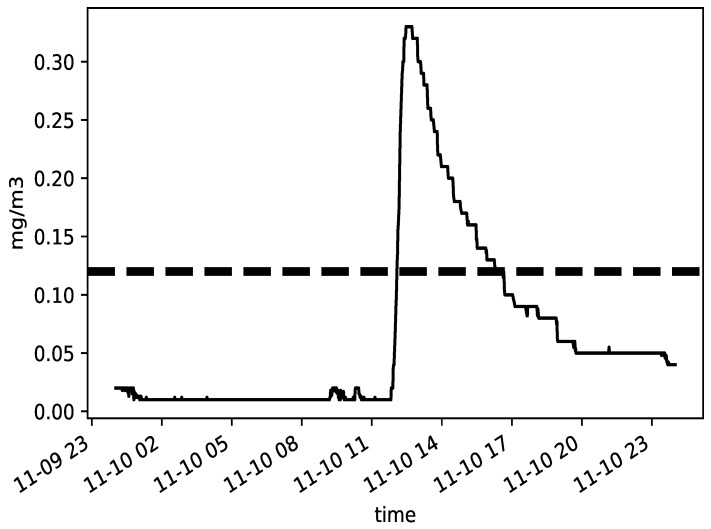
Time evolution of formaldehyde(HCHO) concentration in Site 1 during the day 2018-11-10. Dashed line refers to STEL threshold.

The Occupational Safety and Health Act of 1970 (OSHAct) was passed to prevent workers from being harmed at work. The act created the OSHA, which enforces protective workplace safety and health standards. To fulfill legal compliance, employers used to contact accredited inspectors to perform air-quality testing. However, as presented in Table 2, some standards are defined to help regular short-term sampling. For example, the sampling duration time (15 min) is in relation to minimum 4 times samples established by statistical analysis and practical experience. However, it is still challenging for inspectors to select a sampling period. IAQ varies as working condition change such as different working process. As shown in Figure 4, heave printing work was done in afternoon in Site 1 leading to formaldehyde concentration rising and reaching to peak.

Practical research was conducted in Site 1, which has proved that regular spot checks are not sufficient to ensure employee health and safety. Suppose the inspector implemented a formaldehyde measuring path of 15 min sampling with stops of 30 min in between. If the inspector starts the testing by 09:00 a.m. considering that employee starts working at 08:00 a.m. the sampling period covers from 09:00 a.m. to 11:00 a.m. As depicted, formaldehyde evolution for the 10th of November in 2018 in Site 1, shown in Figure 4, there would not be any exceeding found. However, if the time span is expanded wider, exceedings appear continuously between 12:00 p.m. to 17:00 p.m. Therefore, it is proven that the inspector criteria, in full respect of regulation, still fails to guarantee employee health and safety. Although some surveys could be conducted, such as a review of work patterns, production processes, and exposure times that help select better sampling periods, they are always time-consuming and less accurate.

**Figure 5:**
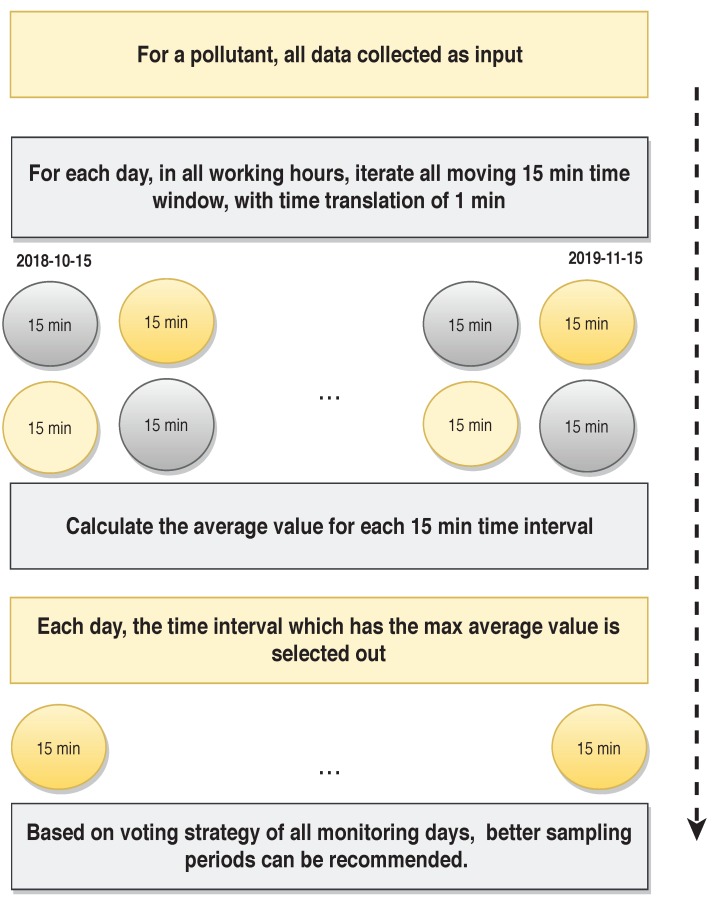
Recommendation strategy flow diagram.

**Figure 6:**
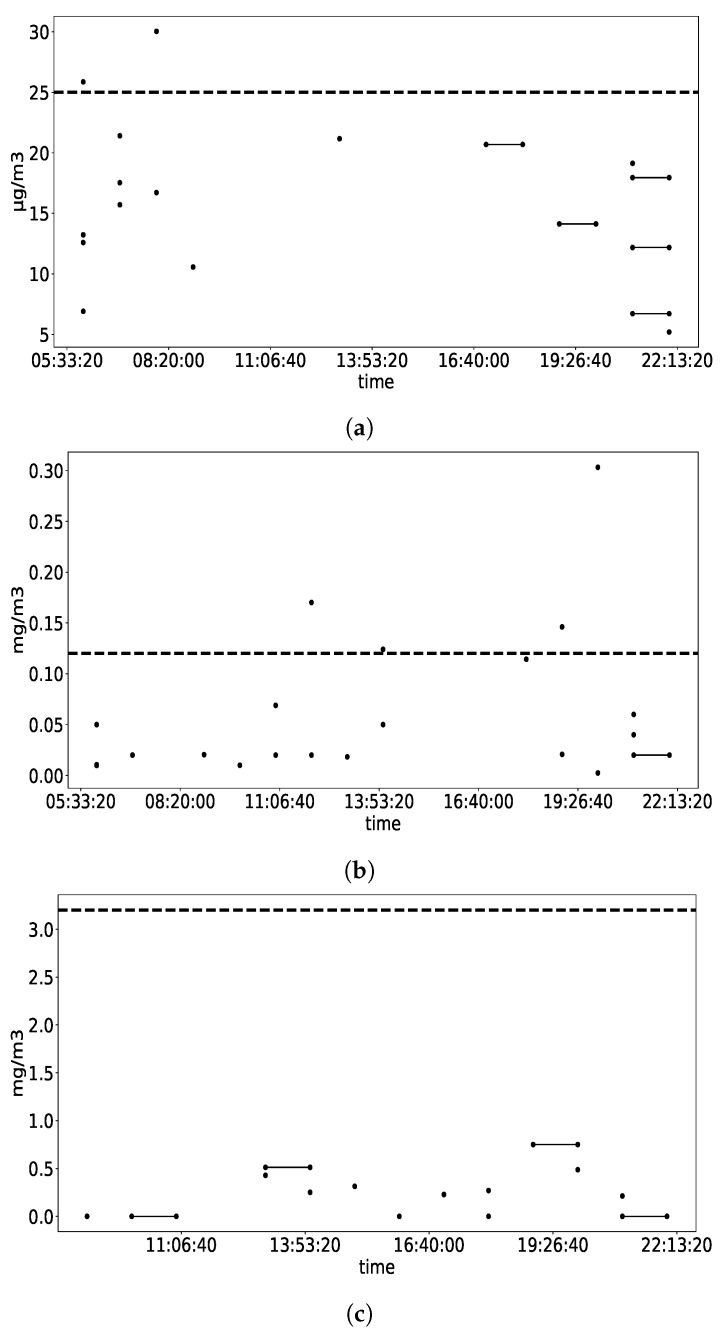
Distribution of indoor pollutant maximal average value occurrences (sampling time: 15 min) in site. (**a**) Distribution of PM2.5 maximal average value during monitoring period in Site 2. Dashed line refers to EPA TLV; (**b**) Distribution of HCHO maximal average value during monitoring period in Site 1. Dashed line refers to STEL threshold; (**c**) Distribution of C_6_H_6_ maximal average value during monitoring period in Site 1. Dashed line refers to STEL threshold.

To address the sampling period selection bias, we designed a recommendation strategy which could give out a reasonable sampling period based on long-term measuring data and a statistical analysis approach. Considering all the historical data collected in the site for each indoor pollutant, a holistic statistical method was used to select better sampling periods. The recommendation strategy working flow is shown in Figure 5. The example of showing how the valid sampling duration for pollutant formaldehyde in Site 1 is selected based on the recommendation strategy is given. The data measurement from the whole data-monitoring period (15 October 2018–15 November 2018) are applied as input data. For each day in the monitoring period, all possible 15 min time intervals daily, with a time translation of 1 min are segmented, and the avg formaldehyde value during each segmentation is calculated. The 15 min segmentation with the max avg value is selected out in each day and the hour and two hours where the 15 min is located is marked and recorded, as shown in Figure 6b. Finally, based on a voting strategy of all monitoring days, those valid sampling durations could be recommended and selected out. Then, specific professional determination methods are used to measure indoor contaminant levels where the time windows are better selected.

The recommendation strategy is evaluated with the case studies conducted in Site 1 and Site 2. Taking PM2.5 in Site 2 as an example, our recommendation framework enables us to demonstrate a distribution of PM2.5 maximal average value occurrences (sampling time: 15 min) in site. The detail is shown in Figure 6a. Each point representing the hour where the better 15 min is located in and each line segment representing the 15 min sampling duration covering 2 h, e.g., from 18:50 to 19:05. As shown in Figure 6a, most high PM2.5 values appear in the morning, before 08:00 a.m. Therefore, based on long-term measuring data analysis, for a regular PM2.5 check in Site 2, the sampling time period is recommended for the morning, before 08:00 a.m. As shown in Figure 6b, most of high formaldehyde values in Site 1 appear in the morning, between 08:00 a.m. to 12:00 p.m. Similarly, as shown in Figure 6c, the recommendation sampling time for benzene in Site 1 would be in the afternoon, around 14:00 p.m. to 18:00 p.m. With the recommendation strategy, exceeding and significant variation could be caught up to ensure employee’s health and safety.

### 3.2. Long-Term Monitoring Benefits for Regulating Working Conditions

**Figure 7:**
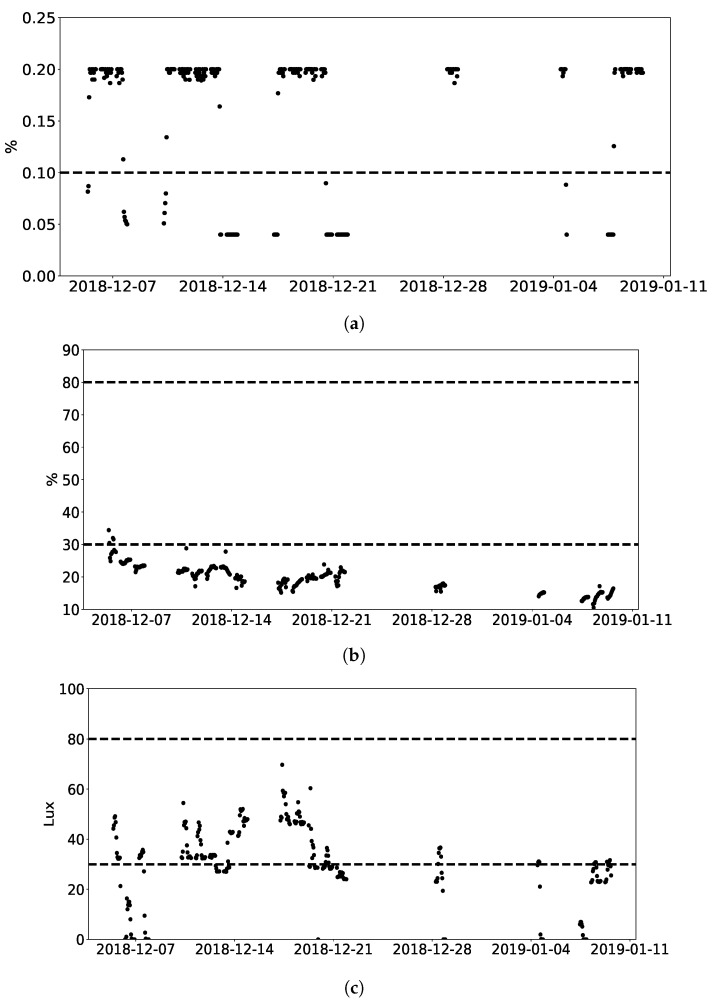
Working conditions demonstration in site 2. (**a**) Evolution of CO_2_ hourly average concentration during monitoring period in site 2. Dashed line refers to Circulate TLV; (**b**) Evolution of humidity hourly average during monitoring period in site 2. Dashed line refers to WHO comfort recommendation; (**c**) Evolution of illumination hourly average during monitoring period in site 2. Dashed line refers to EU illuminance standard.

The employer is legally responsible for ensuring the good working conditions of employees. Long-term IAQ monitoring enables employers to understand IAQ data and IAQ patterns, so they can take appropriate measures to regularly improve working conditions. As shown in Figure 7a, in Site 2, CO_2_ concentration is constantly higher than TLV(0.1%). The TLV(0.1%) is based on a daily average, which obtained references from Circulate App: EnvCon [35]. The Circulate company set TLVs, taking references from China’s air-quality standards. Measures should be taken to reduce CO_2_ concentration in Site 2. The corresponding measures could be opening windows, adapting heating, ventilation, and air conditioning (HVAC) systems or with plants [36,37] to reduce CO_2_ concentration.

Thermal comfort normally refers to temperature and humidity, and is the condition of mind that expresses satisfaction with the thermal environment and subjective evaluation (ANSI/ASHRAE Standard 55). The temperature and humidity ranges are 16–28° and 30–80% respectively, according to WHO. It is also important not to over-design illumination, which can induce adverse health effects such as headache frequency, stress, and increased blood pressure. For the work requiring perception of details, such as offices, sheet metal work, and bookbinding, the minimal illuminance is 100 lux, which is defined by EU standard. On Site 2, as shown in Figure 7b,c, displays very low humidity and illumination levels. Therefore, measures should be taken to improve employee comfort.

### 3.3. Long-Term Monitoring Benefits for OSH Transparency

Long-term IAQ monitoring ensures that OSH dimension is given the same emphasis as other business objectives. The economic benefits of high-quality indoor working conditions was demonstrated by [5]. The magnitude of productivity gains may be obtained by providing better indoor environments, a 20 to 50% reduction in sick building syndrome, saving between 10 and 100 billion US dollars. 8 to 25% fewer asthma-related absences save 1 to 4 billion US dollars. A 23 to 76% reduction in respiratory diseases saves 6 to 14 billion US dollars. The IAQ has a significant effect on economic benefits in enterprise management.

In building science studies, thermal comfort has been related to productivity and health. Office workers who are satisfied with their thermal environment are more productive [38]. For example, it is valuable to provide the correct light intensity for each task or environment. Otherwise, energy not only could be wasted, and over-illumination can lead to adverse health and psychological effects. Beyond the energy factors being considered, glare or excess light can decrease worker efficiency. The field of OSH comprises a variety of risks that need to be managed. Considering economic loss deriving from poor air quality, it is important to report IAQ daily and take it as a Key Performance Index (KPI) vector, along with other business objectives. Moreover, the relevance of the KPIs increases when they are based on real-time measurements.

To quantify how a workplace affects productivity, creativity, and well-being, CBRE, a real-estate services and investment company, designed a science-based tool to measure specific criteria [39]. The related experiments proved that information awareness also affects employee performance. That is to say, if an employee knows they are working in a good quality environment, it is helpful to improve their performance. The real-time long-term monitoring solution would provide IAQ transparent employee empowerment.

### 3.4. Long-Term Monitoring Benefits for Data Sharing by IOTA

Recently, with the tremendous development of Industry 4.0, DLT (e.g., IOTA) has attracted significant attention. With DLT, we will have the Internet of value. DLT has great potential to create new foundations for our economic and social systems by efficiently establishing trust among people and machines, reducing cost, and increasing the use of resources [40]. IOTA, as the most prominent distributed ledger project, whose goal is to become the very fundamental layer of such society, is challenging the looming paradigm shift.

Both continuous collected IAQ raw data and statistical summary data are transmitted to IOTA’s Tangle through MAM or message. Different clients can register their interest to receive such data streams. According to relevant business models, different sharing mechanisms can be packaged to better serve all stakeholders [41]. For example, the calculated avg pollutant value during each 15 min, introduced in Section 3.1, can be uploaded and stored in the IOTA Tangle as the statistical summary data. Relevant accredited inspectors could assess this data in advance for better sampling period selections in OSH assessment.

The long-term IAQ monitoring solution could gain economic benefits for enterprises with the support of IOTA, with which enterprises could receive automatic, transparent, and frictionless payments from IAQ data consumers.

## 4. Conclusions

As proven by our study, long-term IAQ monitoring and data analytics have been lacking in the research community and in practice. Under this context, this research has contributed to address identified gaps by designing and testing a framework and a system, which is proven to be an effective solution for all stakeholder needs. For instance, through the monitoring system, managers can take measures to improve indoor working conditions. Some CO_2_ exceeding values were found in Site 2. Managers could apply one basic green plant, an Areca palm (Chrysaidocarpus lutescens), which was discovered by NASA to efficiently remove CO_2_. In addition, this paper proves the enormous potential of the IIoT in the context of Industry 4.0 to contribute in bringing new insight in worker’s environment, both by consistently monitoring such environment and to easily disseminate the collected information with minimum cost and infrastructure requirements. Besides aligning IOTA, a long-term monitoring solution provides continuous values to OSH assessment agencies, supporting IAQ transparency to employee empowerment, and bringing continuous economic value to enterprises through paid data sharing services.

The limitation of this research study is that precision could issue IoT based measurements, but the data-monitoring system could be re-adapted by the conducted professional spot-check by an accredited inspector, as indicated in Figure 2. At each spot-check, more accurate data measurements could be obtained with the professional instruments. The data-monitoring system would calibrate itself with those accurate measurement, therefore, in the long-term, the precision of the data-monitoring solution would be better improved.

The carried-out research results would be applied in CBRE in future work to assess IAQ influences on employee productivity, health, and well-being.

## Figures and Tables

**Table 1:** TLV for pollutant based on NIOSH, EPA and EU air-quality standards. STEL: Short-Term Exposure Limit.

Pollutant	STEL (15 min)	Average over 24 h
CO_2_	30,000 ppm (54,000 mg/m^3^) STEL	
CO	200 pm (229 mg/m^3^) ceiling	
Benzene	1 ppm (3.2 mg/m^3^) ceiling (15 min)	
Formaldehyde	0.1 ppm (0.12 mg/m^3^) ceiling (15 min)	
NO_2_	1 ppm (0.18 mg/m^3^) STEL	
O_3_	0.1 ppm (0.2 mg/m^3^) ceiling	
PM2.5		50 μg/m^3^ (EPA)
PM10		50 μg/m^3^ from EU air-quality standards

**Table 2:** Minimum number of samples in relation to sampling duration:BS EN 689:1996.

Sampling Duration Time	Number of Samples
10 s	30
1 min	20
5 min	12
15 min	4
30 min	3
1 h	2
2 h	1

**Table 3:** Sensor specification.

No.	Sensor Name	Model	Functions	Range
1	PM	KG-PM2	PM2.5, PM10 Concentration Monitor	0–1000 μg/m^3^
2	HCHO	KG-HO2	HCHO Concentration Monitor	0–7 mg/m^3^
3	TVOC	KG-TV2	TVOC Concentration Monitor	0–3 mg/m^3^
4	C_6_H_6_	KG-C62	C_6_H_6_ Concentration Monitor	0–320 mg/m^3^
5	CO_2_	KG-C22	CO_2_ Concentration Monitor	0–0.5%
6	CO	KG-C12	CO Concentration Monitor	0–500 ppm
7	NO_2_	KG-N22	NO_2_ Concentration Monitor	0–20 ppm
8	O_3_	KG-O32	O_3_ Concentration Monitor	0–20 ppm
9	T.H.I.N	KG-TN2	Comfort Monitor (Temperature, humidity, illumination and noise)	T: −40–80°; H: 0–99.0% RH; I: 0–2000 Lux; N: 0–120 dB

**Table 4:** Building site characteristics overview.

Characteristic	Site 1	Site 2
Section	workshop section	office section
Year of construction	35	46
Floor	1	1
Number of occupants	12	8
Total area (m^2^)	200	100
Heating	No	Yes
Ventilation	Natural	Ventilation System
Windows	Single Glazing	Single Glazing
Floor covering	Coating	Coating
Facilities	One solvent printing machine, two caving machine, computers, furniture	Computers, furniture
Cleaning schedule	Once a week	Everyday
Working schedule	Flexible, 24 h, including weekends	Two shifts: 06:00–14:00; 14:00–22:00, only business days
Smoking	Yes	No
Nearby potential pollutant sources	No	No

## Abbreviations

The following abbreviations are used in this manuscript:

DLT	Distributed Ledger Technologies
GDPR	General Data Protection Regulation
IAQ	Indoor Air Quality
IoT	Internet of Things
IIoT	Industrial Internet of Things
OSH	Occupational Safety Health
TLV	Threshold Limit Value
TVOC	Total Volatile Organic Components

## Appendix A

The literature review method is based on a guideline from [42]. The time span of the review is from Jan 1999 to Jan 2019. Only studies in English are considered. The IAQ monitoring and analytic are considered to be a whole, its sub-problems, such as the standalone monitoring system, exposure risk assessment, and micro-environment are not included in the literature review. The data sources include four digital libraries (ACM Digital Library, IEEE Xplore, Springer Link, and Science Direct), hand searching two conferences: the International Society of Indoor Air Quality and Climate (ISIAQ), the International Conference on Indoor Air Quality, Ventilation and Energy Conservation (IAQVEC) in Buildings. The search terms used are indoor (environmental/air), and quality (investigation/research/assessment/monitoring/analysis). After excluding irrelevant studies based on exclusion criteria and an analysis of their titles and abstracts, 65 studies were included based on full text screening, and 7 studies are selected from the reference lists. In the end, 72 studies were used as the final primary studies. Considering the objective of the research work is mainly from time and data-monitoring solution dimensions, the 23 most relevant studies were selected and listed in Table A1, from pollutant (P), time period (T), seasonal (S), data collection (D), and transparency (Tr) aspects, respectively.

**Table A1:** Systematic literature review of IAQ.

Study	P	T	S	D	Tr
[3]	NO_2_, TVOC, PM0.3-10	8h working time in five working days, September 2015	No	Aeroqual 200 (NO_2_, TVOC), Extech VPC300 (PM0.3-10)	No
[10]	PM2.5, HCHO, CO_2_	PM2.5 and CO_2_ entire year, HCHO in four seasons (sampling time: 20 min)	Yes	A: on-line monitoring system with Ikair ( CO_2_) and Yun (PM2.5) sensors B: on-site measurement for HCHO by spectrophotometry	No
[43]	HCHO, CO_2_	4 h between 08:00 AM and 12:00 AM	No	A real-time occupational exposure monitoring system with Grove-HCHO and T6613C (CO_2_)	No
[8]	NO_2_, O_3_ and 29 VOCs	One week between 20 and 27 December 2012	No	Diffusive samplers	No
[11]	34 VOCs, NO_2_,O_3_	Summer: 24 and 28 May 2010; Winter: February 21 and 25, 2011	Yes	Passive samplers	No
[12]	Temperature, humidity, HCHO, C_6_H_6_, C_2_HCL_3_, Pinene, Limonene, NO_2_, CO_2_, CO, PM2.5, VOCs, Radon, O_3_	Monday to Friday, in both non-heating (26/09/2011-14/10/2011) and heating (23/01/2012-10/02/2012)	Yes	Diffusive samplers (HCHO, C_6_H_6_, C_2_HCl_3_, Pinene, Limonene, NO_2_, O_3_); Telair 7001 (CO_2_), aeroQUAL (CO), PM2.5 (Derenda LVS3.1/PMS3.1-15)	No
[9]	PM2.5, PM10, CO_2_, CO, HCHO, and VOCs, O_3_	1 h	No	Lighthouse handheld 3016 (PM, temperature, humidity), WolfSense (CO_2_, CO, VOC and O_3_), htV-M (HCHO)	No
[44]	PAHs	One month in April	No	Passive sampler	No
[45]	VOCs, HCHO, acetone and O_3_	During 4 h with a 40-m frequency	No	PRO-EKOS AT. 401X (HCHO, O_3_), gas chromatograph Voyager (VOCs and acetone)	No
[46]	temperature, humidity, CO, CO_2_, PM10, NO_2_, HCHO, C_6_H_6_ and toluene, bacteria and fungi	3–10 December	No	Passive bubblers (HCHO), passive bubbler (NO_2_), SKC passive sampler (VOCs)	No
[47]	PM, noise, temperature, humidity	May 2009 (hot season) and February 2010 (cold season)	Yes	–	No
[48]	Bacteria, fungi, dust, ammonia, and HCHO	2 h	No	Passive sampler	No
[49]	Eighteen PAHs	28 days (May–June 2014)	No	Passive sampler	No
[50]	PM	Pre-winter (November and early December 2013) and winter season (January and early February 2014)	Yes	MOUDI	No
[51]	17 VOCs	May 2015	No	Passive sampler	No
[52]	TVOC, 13 VOCs, PM2.5, NOx, O_3_	Two weeks (working and non-working days) which starts from early morning (08:00 a.m.) to late evening (20:00 p.m.)during winter season of 2014	No	Model EC 9810 series (O_3_), Model Ecotech Sernious 40 (NOx), Micro IV Single Gas Detector (CO), MiniVol™ TAS (PM2.5), PhoCheck 5000 photo-ionization detector (PID) (TVOC), NIOSH method (VOCs)	No
[53]	benzene, toluene, ethylbenzene m,p-xylene and o-xylene (BTEX)	Winter (from 9 December 2013 to 17 January 2014) and Spring (from 24 March to 17 April 2014	Yes	Passive sampler	No
[54]	PM	Three weeks during the summer, autumn, and winter in 2014 and 2015	Yes	OPS; TSI model 3330	No
[55]	HCHO and C_6_H_6_	45 min	No	Passive samplers	No
[56]	HCHO	Second semester of 2010 and first semester of 2011	No	Passive samplers	No
[57]	VOCs	24 h	No	Passive sampling	No
[58]	Temperature, humidity, fungi, dust, endotoxins, CHO, VOCs, CO_2_, NO_2_	Two seasons: October–Match; April–September	Yes	Radiello passive sampler (CHO and VOCs), Passam Ag passive sampler (NO_2_), Q-Trak (Temperature, humidity, CO_2_)	No
[59]	PM2.5, PM10	During rush hours (8:00 a.m.–12:00 p.m.) for one week per each season from June 2015–June 2016	Yes	Dust-Trak	No
